# Fabrication and biological properties of artificial tendon composite from medium chain length polyhydroxyalkanoate

**DOI:** 10.1038/s41598-023-48075-8

**Published:** 2023-11-28

**Authors:** Tulyapruek Tawonsawatruk, Anuchan Panaksri, Ruedee Hemstapat, Passavee Praenet, Kasem Rattanapinyopituk, Sani Boonyagul, Nuttapol Tanadchangsaeng

**Affiliations:** 1 Department of Orthopaedics, Faculty of Medicine Ramathibodi Hospital, Mahidol University, Thung Phaya Thai, Ratchathewi, Bangkok, Thailand; 2 College of Biomedical Engineering, Rangsit University, Lak Hok, Pathumthani, Thailand; 3 Department of Pharmacology, Faculty of Science, Mahidol University, Thung Phaya Thai, Ratchathewi, Bangkok, Thailand; 4 Department of Pathology, Faculty of Veterinary Science, Chulalongkorn University, Pathum Wan, Bangkok, Thailand

**Keywords:** Biomaterials, Biopolymers, Biomedical engineering, Biomedical materials

## Abstract

Medium chain length polyhydroxyalkanoate (MCL-PHA), a biodegradable and biocompatible material, has a mechanical characteristic of hyper-elasticity, comparable to elastomeric material with similar properties to human tendon flexibility. These MCL-PHA properties gave rise to applying this material as an artificial tendon or ligament implant. In this study, the material was solution-casted in cylinder and rectangular shapes in the molds with the designated small holes. A portion of the torn human tendon was threaded into the holes as a suture to generate a composite tendon graft. The tensile testing of the three types of MCL-PHA/tendon composite shows that the cylinder material shape with the zigzag threaded three holes has the highest value of maximum tensile strength at 56 MPa, closing to the ultimate tendon tensile stress (50–100 MPa). Fibroblast cells collected from patients were employed as primary tendon cells for growing to attach to the surface of the MCL-PHA material to prove the concept of the composite tendon graft. The cells could attach and proliferate with substantial viability and generate collagen, leading to chondrogenic induction of tendon cells. An in vivo biocompatibility was also conducted in a rat subcutaneous model in comparison with medical-grade silicone. The MCL-PHA material was found to be biocompatible with the surrounding tissues. For surgical application, after the MCL-PHA material is decomposed, tendon cells should develop into an attached tendon and co-generated as a tendon graft.

## Introduction

Autograft tendon transplantation is still the most popular treatment for severe damage to the tendon area^1^. However, the disadvantage of such a method has subsequent problems: repeated injuries of patients, infection risk, and surgical failure^2,3^. Tissue engineering techniques are, therefore, an option that is interested in solving such problems^4^. Tendon reconstruction is a treatment method in patients that do not need to lose other tendons to replace damaged parts. Tissue engineering principles involve the design of materials to create a new organ outline based on the self-regeneration of the body^5^. The self-regeneration of tendons is generally lower rate than other organs^6^. Therefore, choosing the suitable materials for creating skeletal may contribute to rehabilitation. The self-healing mechanism occurs from the cells of the damaged area of the tendon. The materials used should be composited to the tendons in that area to maintain the condition of the original tendons.

Creating composite artificial tendons requires ideal materials with specific properties conducive to the reconstruction of tendons. Biodegradation is an essential property of this concept. The material will be slowly decomposed, and the restoration of emerging tendons will not have foreign objects left^7^. The range of composite tendon treatment must act as a replacement for normal tendons. Movement creates a load on composite tendons due to the support of force from muscle contractions. Such activities create an excellent effect in stimulating growth factors, which contribute to the repair and creation of new tendons^8–10^. The biological augmentation and mechanical properties of materials are important for the above processes. Therefore, human tendons have hyper-elastic mechanical characteristics, where the relationship between stress and stress will not be linear, and the ultimate stress of the tendon is 50–100 MPa^11–14^. The appropriate composite tendon should have a mechanical appearance similar to natural tendons^15^. Considering the material used in conjunction with such concepts is very challenging. Hyper-elastic materials are highly flexible materials such as rubber, which are rarely found biodegradation in these materials^16^. In addition, the specificity of the properties as mentioned above makes the advancement of such techniques limited^17^. However, the study of bioplastics has various properties in line with the creation of composite tendons. The bioplastics above are medium-chain-length polyhydroxyalkanoate (MCL-PHA).

The medium chain length polyhydroxyalkanoate or MCL-PHA is a polymer produced and accumulated in *Pseudomonas* strains with a carbon source feeding, then extracted from the microorganisms for further applications^18^. MCL-PHA belongs to the group of elastomer and hyper-elastic polymers like tendons. The material also contains study data on biocompatibility and biodegradability^19^. The above data show that MCL-PHA will likely be used for composite molding with tendons. Moreover, the fabrication of artificial tendons for composites necessitates consideration of the composite shape and appearance, which affect the mechanical properties of the application.

In this study, MCL-PHA artificial tendons are molded in different shapes. Each artificial tendon is punched and threaded together with human tendons to determine mechanical properties. The hole characteristics on MCL-PHA are based on simulations with previous study programs, which simulate the position of the hole with the least accumulated stress in the hole area to reduce the chance of tearing of artificial tendons^7^. The approach of suturing tendons with MCL-PHA is a novel method devised for tendon regeneration. This research introduces the concept of combining the material with tendons in a composite manner to substitute the conventional treatment, which involves surgically attaching tendons from other areas to the torn tendon site. The rationale behind the concept of perforation and suturing is to provide a scaffold material that supports the complete healing of the torn tendon, employing tissue engineering principles. This innovative approach cannot be referenced as a conventional method since the stitching or suturing of tendons for tendon treatment relies on medical judgment and the extent of tendon damage. This study aimed to prove that the composite of the tendon with the material can share the load on the joint and have the same strength as the average human tendon. In addition to the above, fibroblast cell adhesion tests on molded MCL-PHAs were carried out. Furthermore, cell compatibility and collagen type 1 synthesis were tested when fibroblast cells were cultured with MCL-PHA artificial tendon to prove that the material does not inhibit collagen type 1 synthesis. Besides, the safety biocompatibility evaluation of the local tissue response to MCL-PHA artificial tendon was further investigated in a rat subcutaneous model compared to a medical grade reference material.

## Experimental

### Fabrication of MCL-PHA artificial tendon

The MCL-PHA used in the study was produced on a pilot scale based on previous studies^20,21^. The forming of MCL-PHA uses the chloroform solvent casting method into the mold. The Fabrication characteristics were divided into two forms: cylindrical shape and rectangular shape. The mechanical properties of each structure were tested under various conditions. The rectangular shape was made of Bakelite molds, which form a rectangular cavity measuring 10 × 40 × 50 mm. The cylindrical shape uses a silicone tube mold; the internal size is ⌀5 × 30 mm. The mold height exceeds the actual specimen because mixing the material with the solvent increases the volume. The exact dimensions of the rectangular and cylindrical pieces are 10 × 40 × 50 and ⌀5 × 40 mm, respectively. The thickness and diameter of the specimen are based on the average human hand tendon size^22^. MCL-PHA dissolved in chloroform (5 mL for cylindrical and 50 mL for rectangular) was poured into prepared molds. The amount of MCL-PHA used for molding is calculated from Eq. (1). The solution in the mold evaporated at room temperature in two days for a cylinder and four days for a rectangular shape. The dried part was removed from the mold by destroying the mold.

1 $$MCL-PHA\,quantity\,for\,casting=\left(V\times d\right)$$

where V is the required volume of the specimen, and d is material density (MCL-PHA = 1.25 g/cm^3^)

### Human samples declaration

All the human samples in this study were approved by the Human Research Ethics Committee (COA. MURA2020/2011). We confirm that all methods and experimental protocols were carried out in accordance with relevant guidelines and regulations. Human sample used for this experimental study was obtained from the Department of Orthopaedics, Faculty of Medicine Ramathibodi Hospital, Mahidol University as the waste sample left over specimen with the ethical committee approval. However, the demographic data are not allowed to be tract to the patient profile.

### Mechanical properties test of MCL-PHA artificial tendon composite

**Figure 1:**
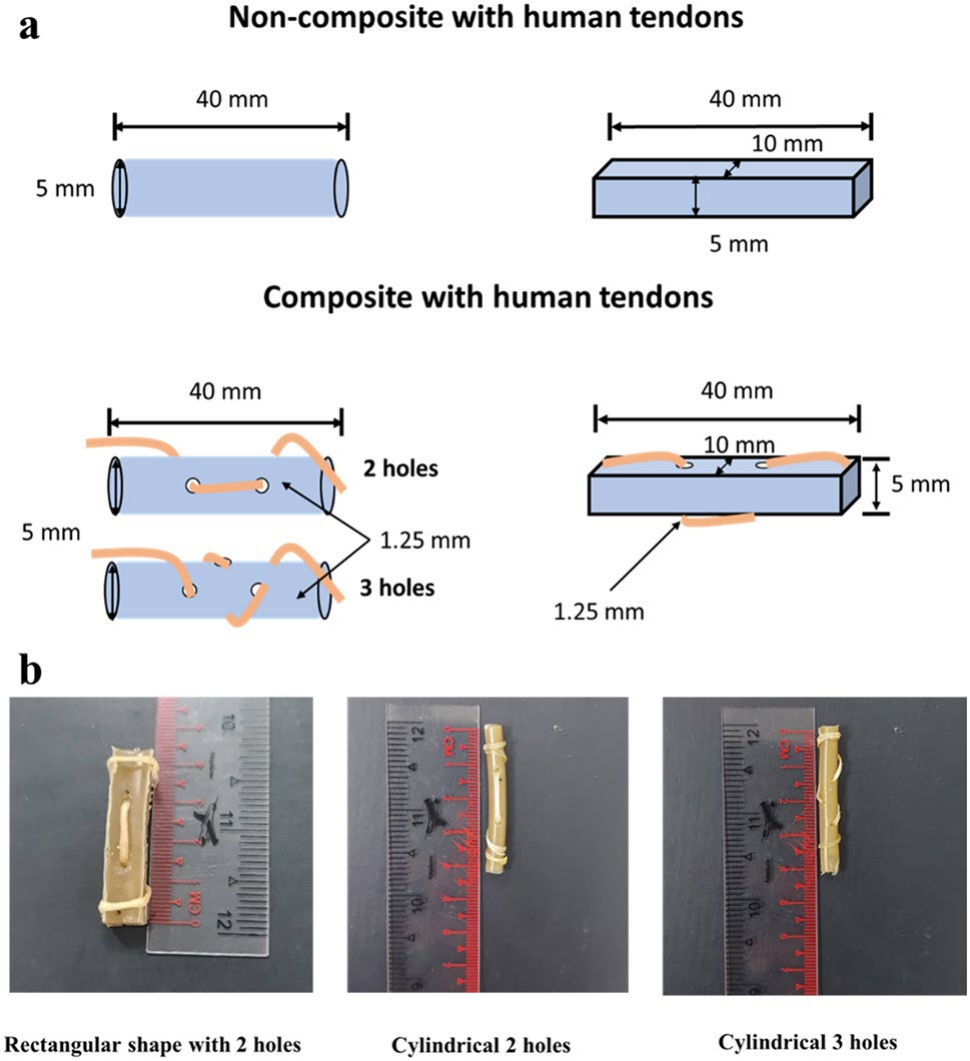
(**a**) Different shapes (rectangle versus cylindrical) of MCL-PHA tested for mechanical properties (**b**) Characteristics of human tendon stringing with molded MCL-PHA.

The MCL-PHA artificial tendon used in the study was divided into two conditions: non-composite with the human plantaris tendon (fresh samples at room temperature) and conditioned composite with the human plantaris tendon, as shown in Fig. 1a. The composite of the human tendon to the specimen is punched on both rectangular and cylindrical specimens. The bore size is the same at 1.25 mm, based on simulations of mechanical forces on the perforated artificial tendon, resulting in minimal breakage^7^. The number of holes drilled is equal to 2 in both shapes, but the cylinder will increase the state of 3 holes to observe the nature of the side hole threading and the increase of the stringing point. Sample sizes and test conditions are summarized in Table 1. The sample length was cut into three parts of the molded specimen for retesting (n = 3). The human tendon used in the composite is the Plantaris tendon, which is similar in size to the hand tendon to be studied. The whole test pattern is shown in Fig. 1a, with the attachment of the sample to the Plantaris tendon as shown in Fig. 1b. The samples were tested with a tensile testing machine (Universal, Ibertest, Spain) at a distance of 20 mm, and the test speed was 100 mm/min. The composite sample is considered as a single material because it has been proven that there will be no force accumulation at the point of tendon stitching. The gripping area was reinforced with sandpaper to achieve a dumbbell shape to avoid breaking at non-central points of the sample. The paper increases the surface area at the gripping head and locks the material onto the gripping head. The cross-sectional area of the composite sample is calculated based on the cross-sectional area of the formed MCL-PHA material. Even if there are holes in the material, they are replaced by the stitched tendon, so the composite is considered to have a similar cross-sectional area. The test set a preload value equivalent to 0.1 N from the buckled state while preconditioned at 8% strain. The data from the tests will be used to plot the relationship between strain and stress, from which the Young’s modulus is calculated from the curve of the graph. For elastomer materials, the curve properties are non-linear or discontinuous. This study only considered the slope of the graph before the point of linearity or before the slope changes.

**Table 1:** Design parameters of MCL-PHA artificial tendon for mechanical testing.

Shape	Sample size (mm)	Number of holes
Cylindrical	40 × ⌀5	0
		2
		3
Rectangular	40 × 10 × 5	0
		2

### Cell culture preparation

The human fibroblast cells were taken from paediatric dermal skin. The fibroblast cells were grown in Dulbecco's modified eagle medium (DMEM; Gibco, MA), supplemented with 10% fetal bovine serum (FBS, Gibco, MA) and penicillin (100 U/ml)/streptomycin (100 µg/ml) (Gibco, MA). Cells were maintained at 37 °C in an atmosphere of humidified air with 5% CO_2_.

### Sterilization and preparation of MCL-PHA artificial tendon for testing on fibroblast cells

The MCL-PHA artificial tendon was sterilized by soaking in 70% ethanol for 5 min, rinsed three times with phosphate-buffered saline (PBS; Millipore, MA), air drying in laminar airflow, and exposed to the ultraviolet (UV) light for 40 min. The preparation of MCL-PHA samples for cell viability testing involves an indirect treatment. The MCL-PHA samples with varying amounts are immersed in culture medium and incubated for 3 days to extract the MCL-PHA into the medium. The final concentrations of MCL-PHA extracts in culture medium are 1, 10, and 100 mg/ml, which will be used for cell viability testing. A direct treatment method will be used to prepare samples for cell adhesion testing. This involves treating MCL-PHA artificial tendons directly with fibroblast cells.

### Cell attachment

MCL-PHA artificial tendon was prepared for the adhesion of fibroblast cells on the surface. MCL-PHA specimen (cylindrical size ⌀5 × 40 mm.) was sterilized and put on the cell culture dish (⌀100 × 17 mm, Thermo Fisher Scientific, USA). Fibroblast cells were seeded at 2 × 10^6^ cells/dish density and incubated for seven days. Changing the culture medium was performed every two days during the incubation period. The specimen was taken from the cell culture dish to prepare for fixation. First, the MCL-PHA sample was washed with phosphate-buffered saline (1x, pH 7.4). Then, MCL-PHA and cells were fixed with 4% paraformaldehyde for one hour. The MCL-PHA artificial tendon is cut from the surface of the specimen for examination via a confocal microscope (Olympus FV3000, Japan). The samples were stained with DAPI Staining Solution (Sigma-Aldrich, MA) (1 mg/ml concentration) in a dark room. Cell adhesion on the specimen surface was observed with Olympus confocal microscope under × 10 magnification. The above MCL-PHA samples were compared with cells without MCL-PHA and MCL-PHA that were not co-cultured with cells.

### Cell viability

The MCL-PHA artificial tendon was determined on cell viability by MTT assay. Fibroblast cells were suspended and seeded 100 µl in 96-well plates at 5 × 10^3^ cells/well density and incubated overnight. Cells were treated with varying concentrations of the MCL-PHA artificial in culture medium at 1, 10, and 100 mg/ml for 24 h. 3-(4,5-dimethylthiazol-2-yl)-2,5-diphenyltetrazolium bromide solution (MTT; Biobasic, UK) was prepared by dissolving with PBS at 5 mg/ml for 100 µl in each well, followed by 100 µl of culture medium as a final concentration of 2.5 mg/ml, and then incubated for one hour. Dimethyl sulfoxide (DMSO; Sigma-Aldrich, MA) was used for dissolving the formazan crystals formed, and the quantity of the colored formazan derivative was determined by measuring absorbance at 570 nm with 620 nm as a reference filter. The experiment was performed in three replicates, and the percentage viability was calculated as % viability = [OD of treated cells/OD of control cells] × 100.

### Measurement of collagen type 1

MCL-PHA samples were incubated with human fibroblast cells for seven days. Protein extraction was performed using RIPA buffers for assays. In preparation for the test, total protein content was measured with the Bradford reagent kid (Bio-Rad Protein Assay). Collagen type 1 measurement from extracted proteins was performed through the human collagen type 1 Elisa kit (My BioSource, CA). The samples prepared with the assay were measured at an optical density of 450 nm to calculate the amount of collagen type 1.

### In Vivo biocompatibility testing

#### Experimental designs and sample implantation

**Figure 2:**
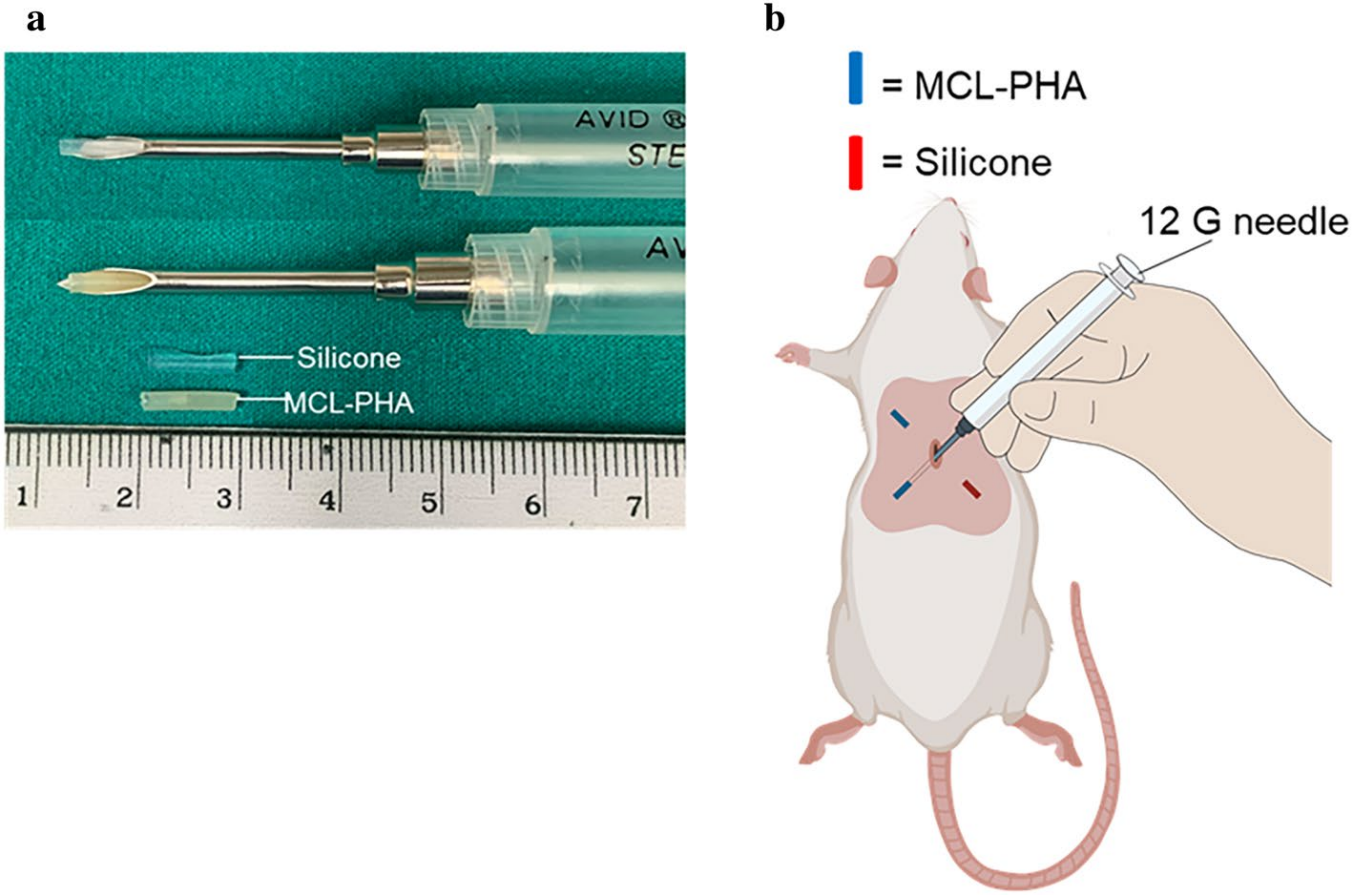
(**a**) Loaded implant material into the lumen of the 12-gauge needle. (**b**) Four positions of subcutaneous implantation in a rat.

Seven to eight weeks old male (200–230 g) Wistar rats (Nomura Siam International Co., Ltd., Bangkok, Thailand) were purchased and housed in an Association for the Assessment and Accreditation of Laboratory Animal Care (AAALAC) accredited facility. The experimental protocol was performed in accordance with relevant guidelines and regulations. This study is reported following ARRIVE guidelines (Animal Research: Reporting of in vivo Experiments; https://arriveguidelines.org) and was approved by the Institutional Animal Care and Use Committee (IACUC) of the Faculty of Science, Mahidol University (Protocol No MUSC65-014–607). Nine animals were randomly allocated into three experimental groups (n = 3/group) according to study endpoints as follows: group I: 7 days, group II: 14 days, and group III: 28 days. Each animal was subjected to subcutaneous implantation with test material (MCL-PHA) in comparison to control material (medical-grade silicone; Neoplastomer Co., Ltd, Thailand). In brief, rats were anesthetized by isoflurane (Attane™) inhalation (5% for induction; 2–3% for maintenance). The dorsal area of the rat was shaved and cleaned with povidone-iodine. The implantation was performed under anesthesia, in which a stab incision 2 mm in length was created in this procedure. After loading the implant material in the lumen of the 12-gauge needle, the needle was inserted through a stab incision into the required position and depth. With the plunger needle, the MCL-PHA or silicone was pushed out and deposited in the subcutaneous tissue of both sides of the dorsal area. Two pieces of MCL-PHA were inserted on the left side, while on the right side was inserted with 2 pieces of silicone (Fig. 2). The tunnel was closed with a stitch of the suture.

### Histopathology examination and Scoring of the implantation site

After the animals were euthanized by an overdose of thiopental sodium (Anesthal®) (100 mg/kg body weight; i.p.), the tissue samples, including implant together with sufficient surrounding tissue, were collected to enable evaluation of the local histopathological response. Tissue samples were preserved in 10% neutral-buffered formalin for 2–3 days and then embedded in paraffin wax. For histological examination, paraffin-embedded skin tissue sections were cut at 4 µm and stained with hematoxylin and eosin (H&E). An experienced pathologist examined sections under a light microscope in a blinded fashion. The histological scoring system was followed by the ISO 10993-6:2016 Biological evaluation of medical devices Part 6: Tests for local effects after implantation (International Organization for Standardization, 2016), shown in Table 2. Each sample was randomly selected 5 to 7 high-power fields for scoring.

**Table 2:** Histological lesion scoring criteria, modified from ISO 10993-6:2016 Biological evaluation of medical devices Part 6: Tests for local effects after implantation.

Histological features	Score				
	0	1	2	3	4
Hemorrhage	None	Mild	Moderate	Severe	-
Inflammatory response; polymorphonuclear cells, lymphocytes and plasma cells	0	Rare, 1 to 5 cells/HPF	Rare, 5 to 10 cells/HPF	Heavy infiltration	Marked infiltration (Packed)
Foreign body response (Macrophage and multinucleated giant cell infiltration)	0	Rare, 1 to 2 cells/HPF	Rare, 3 to 5 cells/HPF	Heavy infiltration	Marked infiltration (Sheet)
Fibrosis	None	Narrow band	Moderately thick band	Thick band	Extensive band

### Statistical analysis

One-way ANOVA using SPSS software was used to analyze the difference in cell viability from the MTT Assay. In addition, the Independent Sample T-test was used to analyze the difference in the amount of collagen type I to total protein content between normal cells and incubated cells with MCL-PHA. Independent sample t-test is also used to analyze the ultimate strength of each composite tendon closest to human tendons (confidence interval of 95%, P < 0.05). GraphPad Prism version 6.0.1 (GraphPad Software, La Jolla, CA, USA) was used for the in vivo biocompatibility testing and histopathology scoring. Scoring data were averaged and expressed as the standard error of the mean (SEM) values by using the Mann Whitney U test. P values less than 0.05 were considered statistically significant.

### Institutional review board

The animal study was approved by the Institutional Animal Care and Use Committee (IACUC) of the Faculty of Science, Mahidol University (Protocol No MUSC65-014-607). All the human samples in this study were approved by the Human Research Ethics Committee of the Faculty of Medicine Ramathibodi Hospital, Mahidol University (COA. MURA2020/2011). We confirm that all methods and experimental protocols were carried out in accordance with relevant guidelines and regulations.

### Informed consent

All study participants, or their legal guardian, provided informed written consent prior to study enrolment.

## Results and discussion

### Fabrication of MCL-PHA artificial tendon

The MCL-PHA artificial tendon produced through fabrication has the characteristics of a complete specimen that matches the size of the designed mold. The specimen can be observed in Fig. 1b. Creating different shapes is based on the shape of the tendon, which is indeterminate^23^, affect different mechanical properties. However, the rectangular design uses a larger amount of MCL-PHA than the cylinder compared to the same length. The reason for not drilling additional holes in the square shape is that the 3-hole drilling condition of a square could not be compared with the 3-hole drilling condition of a cylinder because it would make the length of the tendons used in the stringing unequal. The 2-hole drilling could therefore be used for comparison because it pierces in the same plane allowing control of the sample conditions. However, drilling 3 holes in a cylinder increases the number of holes in the lateral plane of the piece by 1, performed merely for the cylinder to compare the difference between drilling 2 holes and 3 holes in a cylinder.

### Mechanical properties of MCL-PHA artificial tendon

**Figure 3:**
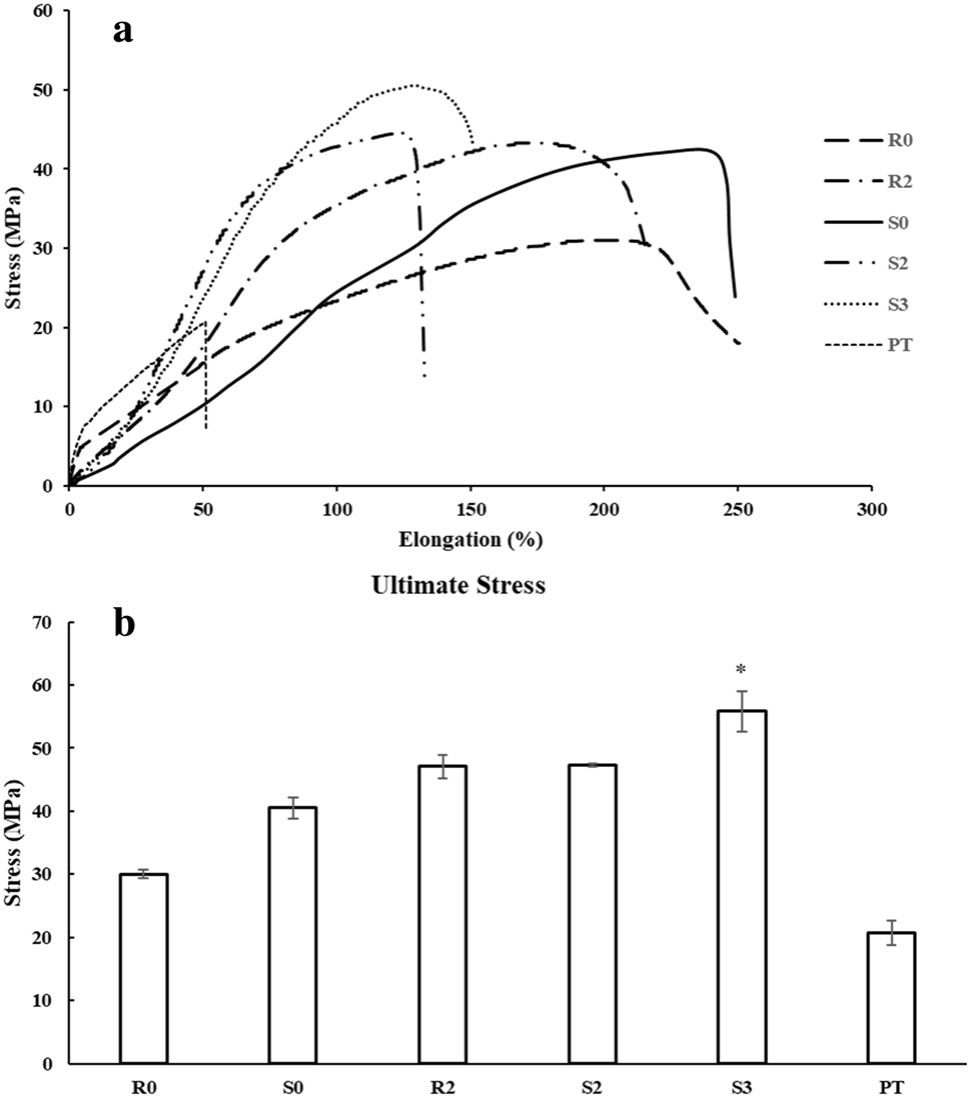
(**a**) The relationship between the stress value and the percentage elongation of the MCL-PHA artificial tendon under each test condition (**b**) Ultimate strength for each test condition (n = 3, p < 0.05, *significant, error bars of standard deviation).

The MCL-PHA artificial tendons with different shapes and conditions are tested for mechanical properties to find the right conditions. Table 3 shows the ultimate strength values, Young's modulus, and elongation at break (%) of each condition sample (n = 3). The ultimate strength of MCL-PHA composites with tendons (cylindrical and rectangular shapes) is greater than non-composite MCL-PHA. The 2-point (hole) tendon composite at different shapes has a similar ultimate strength at 47 MPa. The tensile testing revealed a failure characteristic of the samples occurring at a similar location, specifically near the midpoint of the specimen. However, the composite samples displayed non-simultaneous failure of the material components. The difference in modulus between the material and the tendon is significant, leading to the material breaking first, with the test continuing until the tendon eventually breaking later. This failure behavior is observed in every form of the composite samples. The above results show that the designed shape does not affect the ultimate strength. The stress of the material is caused by force exerted on the cross-sectional area of the material, and the cross-sectional area of the tendon design shape is not very different^24^. Figure 3a shows the relationship curve between the elongation and the stress of the MCL-PHA artificial tendon under various conditions. The relationship curve indicates the reduced elongation of MCL-PHA artificial tendon when the elongation at break (%) of two shapes MCL-PHA artificial tendon is equal to 132 to 154%. The decrease in elongation and the increase in the ultimate strength of MCL-PHA artificial tendon composites may occur due to load sharing of tendons together with MCL-PHA. The above hypothesis is performed with the design of a 3-holes cylindrical MCL-PHA tendon composite. The results show that the 3-holes MCL-PHA tendon composite has more ultimate strength than a 2-hole MCL-PHA tendon composite. An increase of hole drilling on the material should reduce the stress, but the composite thread to the material results in load sharing, which increases ultimate strength^25^. The ultimate strength of each condition is comparable to the ultimate strength of human hand tendons (50–100 MPa)^26^. Figure 3b shows that the 3-holes composite MCL-PHA artificial tendon has the strength value closest to the human hand tendon, which is 55 MPa. The mechanical properties of the materials used to create new tendons are very important. The material's strength must be considered first, which must have properties as close to human tendons as possible. MCL-PHA is a material that tends to be used as a composite scaffold for tendons due to its potential to provide load-bearing support. This is attributed to the fact that the material's ultimate strength is such that it does not significantly differ from the ultimate strength that causes tendon rupture. Although there may be differences in modulus between the material and the tendon, the stress that leads to sample failure is quite similar due to variations in the strain proportions. However, testing results could hypothesize that both the material and the tendon might experience load sharing. This stems from the non-linear nature of the stress–strain relationship graph between stress and strain for both the tendon and MCL-PHA. This characteristic, typical of elastomeric or rubber-like materials, suggests that the non-linear nature of the sample's mechanical properties prevents making definitive assumptions about load sharing, even though the fracture resistance of the samples may differ. Beyond the material testing, where MCL-PHA failed before the plantaris tendon, despite the higher failure point of the MCL-PHA, it can be inferred that reinforcement might occur or load sharing could take place within the MCL-PHA composite scaffold. Moreover, the shared load may occur in a way that the material bears a higher burden due to its failure before the tendon in the composite sample. The failure behavior of the composite sample might arise from pulling the material, which could cause an imperfect bond between the material and the tendon. This results in the majority of the load being borne by the material with a lower modulus, leading it to fracture before the tendon. However, such behavior increases the mechanical properties.

**Table 3:** Mechanical properties of MCL-PHA artificial tendon with different shapes and composite characteristics (n = 3, ± standard deviation).

Condition	Mechanical properties		
	Young's modulus (MPa)	Elongation at break (%)	Ultimate strength (MPa)
Rectangle without holes (R0)	36.46 ± 5.36	176.80 ± 14.05	30.04 ± 0.61
Rectangular composite tendon (2 holes) (R2)	36.66 ± 5.98	154.91 ± 36.50	47.26 ± 1.83
Cylindrical without holes (S0)	29.05 ± 4.08	215.02 ± 16.64	40.50 ± 1.73
Cylindrical composite tendon (2 holes) (S2)	27.46 ± 2.68	132.27 ± 16.18	47.10 ± 0.24
Cylindrical composite tendon (3 holes) (S3)	64.91 ± 4.02	153.00 ± 13.11	55.83 ± 3.16
Plantaris tendon (PT)	198.74 ± 7.51	50.74 ± 7.24	20.69 ± 1.90

### MCL-PHA artificial tendon test in combination with fibroblast cells

**Figure 4:**
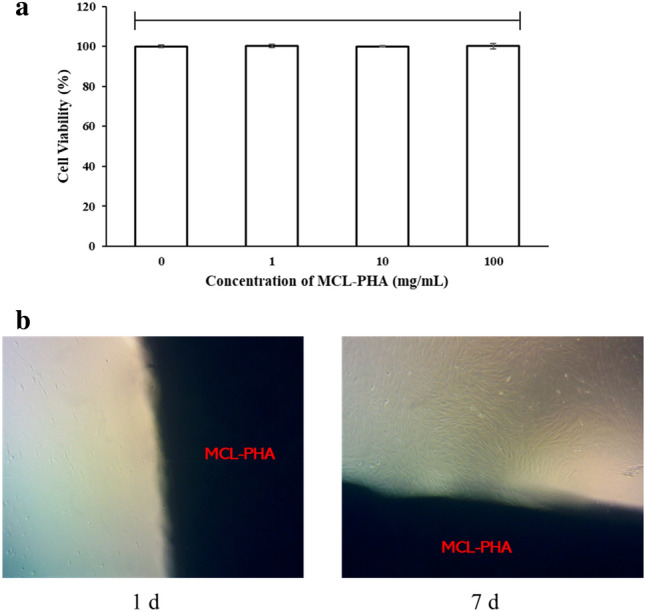
(**a**) Cell viability at MCL-PHA concentrations of 0 mg, 1 mg, 10 mg, and 100 mg (n = 3, p < 0.05, ns = non-significant, error bars of standard deviation) (**b**) Cells with MCL-PHA (black shadow) for 1 day (1d) and 7 days (7d) via invested microscope at 4X magnification.

The effect of MCL-PHA artificial tendon on fibroblast cells is assessed to observe cell adhesion and cell viability test. Fibroblast cells are the main component of tendons^27^. Proving that such cells can adhere to MCL-PHA artificial tendons, in which it is essential for tendon regeneration^28^. The survival of fibroblast cells, when cultured together with MCL-PHA, is carried out by MTT assay. The results show cell viability at MCL-PHA concentrations of 1 mg, 10 mg, and 100 mg, respectively (Fig. 4a). The results showed a non-significant difference in cell viability (P < 0.05), with cell viability approaching 100%. An image from an inverted microscope of fibroblast cells on day 1 and day 7 of culture (Fig. 4b) shows that cells can survive and coexist with molded MCL-PHA. The test results correspond to data that reports MCL-PHA has biocompatibility^29^.

### Measurement results of collagen type 1 from fibroblast cells on MCL-PHA artificial tendon

**Figure 5:**
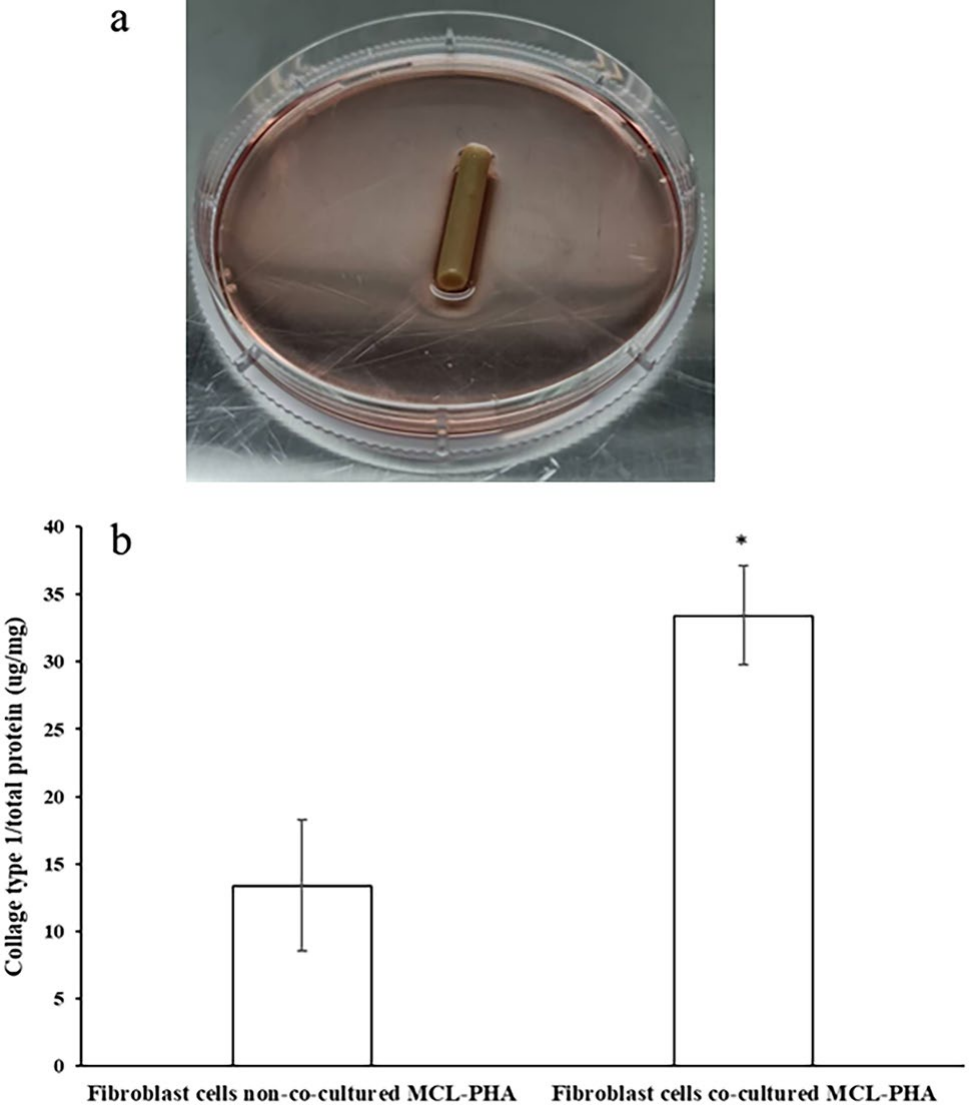
(a) MCL-PHA artificial tendon co-cultured with fibroblast cells (b) Comparison of collagen type 1/total protein content of fibroblast cells co-cultured MCL-PHA and fibroblast cells non-co-cultured MCL-PHA (n = 3, p < 0.05, *significant, error bars of standard deviation).

The measurement of collagen type 1 of fibroblast cells cultured with MCL-PHA artificial tendon is necessary because collagen type 1 is an essential component of tendons. The strength of the tendon comes from collagen, and the amount of collagen type 1 created also indicates the efficiency of tendon restoration^30^. The amount of collagen type 1 is measured from fibroblast cells cultured with MCL-PHA artificial tendon (Fig. 5a). Comparing the results of collagen type 1/total proteins of fibroblast cells that are not cultured with MCL-PHA artificial tendon and fibroblast cells cultured together with MCL-PHA artificial tendon are shown in Fig. 5b. The above results show that collagen type 1 of fibroblast cells cultured with MCL-PHA artificial tendon is higher than fibroblast cells without MCL-PHA artificial tendon. The data proves that MCL-PHA artificial tendon has no inhibitory effect on the increase in the amount of collagen type 1 created by fibroblast cells. A statistically significant increase in collagen type 1 compared to normal fibroblast cells shows that the material stimulates collagen type 1 production to increase strength and regeneration. These *in-vitro* tests show a potential possibility of restoring tendons completely.

### In vivo biocompatibility test of MCL-PHA artificial tendon

**Figure 6:**
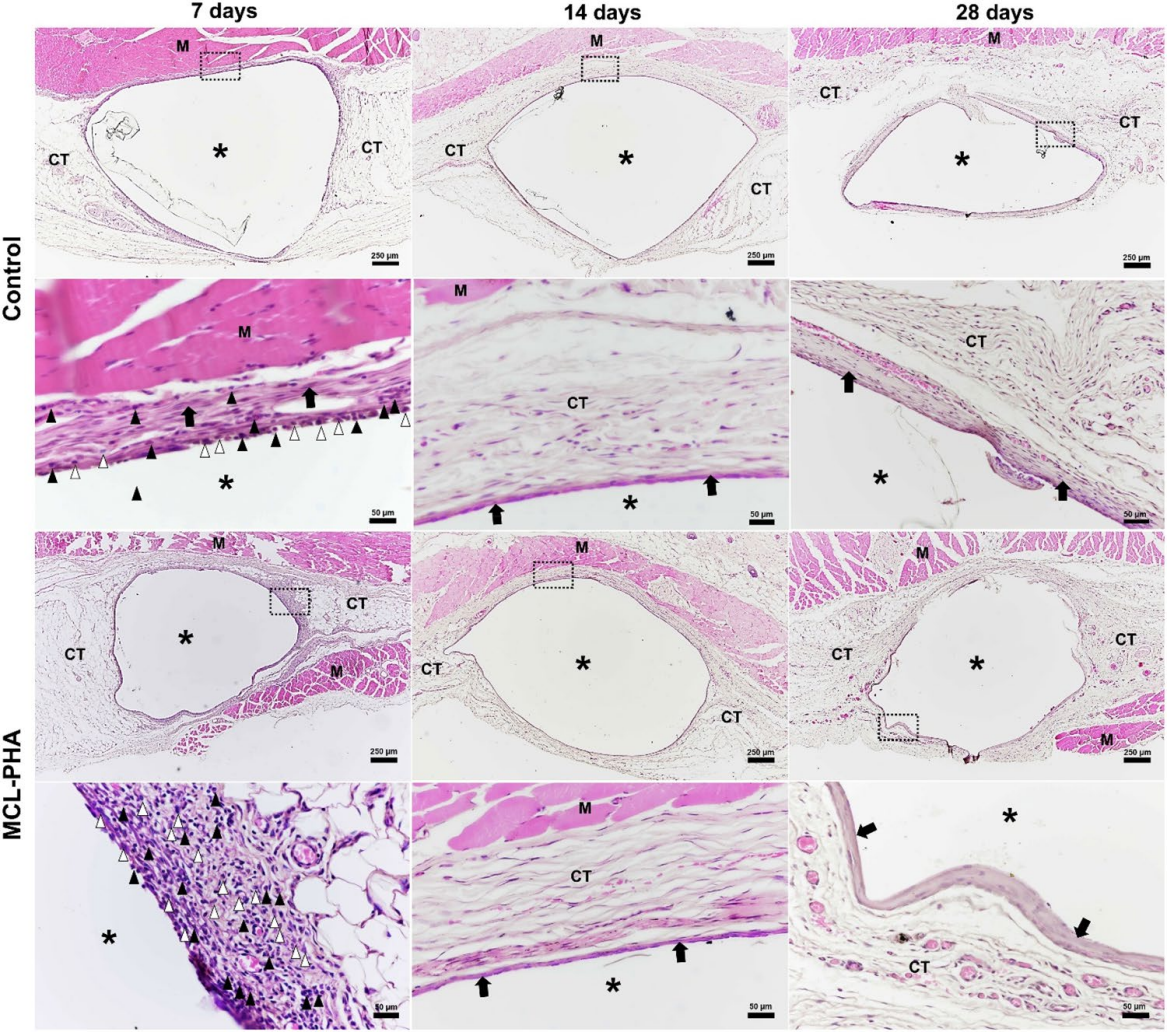
Histological findings of tissue after implantation of materials (asterisks) adjacent to the tendon at days 7, 14, and 28. Fibrosis is demonstrated surrounding the implanted materials (arrows). Inflammatory cell infiltration (black arrowheads), predominated by lymphocytes, plasma cells, and occasionally polymorphonuclear cells, and foreign body reaction, characterized by macrophage infiltration (white arrowheads) are also observed, especially at day 7 after implantation of material (H&E stain). High-magnification figures represent the area in the dashed line box in the low-magnification figures above. *M* muscle, *CT* subcutaneous connective tissue.

Histological findings demonstrated the implanted materials in the subcutaneous layer of a rat model following the implantation at three different periods (Fig. 6).

Surrounding the implanted materials showed inflammatory cell infiltration with dominant lymphocytes, plasma cells, and occasionally polymorphonuclear cells from day 7 to day 28 after implantation (Fig. 6). The highest inflammatory and foreign body responses showed in day 7 after implantation and decreased with time on both control and MCL-PHA groups. A low degree of fibrosis characterized by only a narrow band of fibrous connective tissue surrounding the materials was found in most samples (Fig. 6). In addition, necrosis of the adjacent tissues was not observed in all samples.

**Figure 7:**
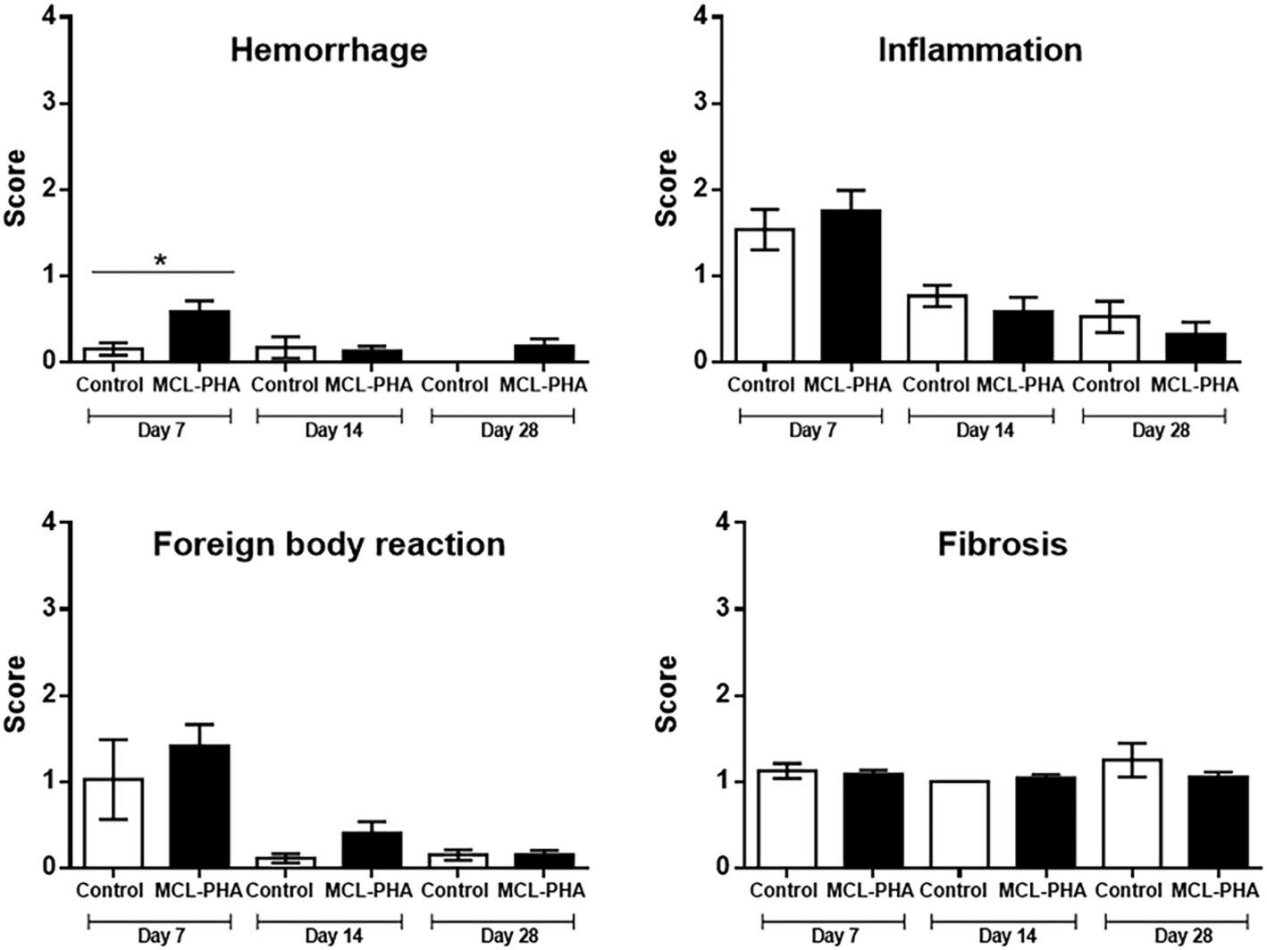
Histological lesion scores according to ISO 10993-6:2016 Biological evaluation of medical devices Part 6: Tests for local effects after implantation. *Indicates the significant difference between implanted material and control (*p* < 0.05), error bars standard deviation.

The semi-quantitative analysis of host tissue reactions was also performed in the subcutaneous layer. Although a significantly higher degree of hemorrhage adjacent to the implanted materials was seen on day 7 after implantation in the MCL-PHA group compared to the control group, this was a mild degree of hemorrhage (Score 0–1), which then declined with time (Fig. 7). The inflammatory and foreign body responses, as well as fibrosis scores, was not significantly different among duration and experimental groups (Fig. 7).

## Conclusions

MCL-PHA artificial tendon molding for composites with human tendons tends to be similar in strength to human hand tendons. In addition, the material and the tendon may share the load of the force resulting in increased strength. Increasing the stringing point gave the most significant strength; the cylinder was considered suitable for the application due to the lower MCL-PHA content required for forming and the strength equivalent to the rectangular shape in the same condition. Cell adhesion tests on molded material showed that cells could adhere to the surface and that the material had cellular compatibility and increased collagen type 1 synthesis. In addition, the MCL-PHA material was found to be biocompatible with the surrounding tissues. The above results demonstrate the possibility that the MCL-PHA material could be further developed and utilized at the clinical trial level and put into practice.

## Data Availability

The data presented in this study are available on request from the corresponding author.
