# Author Correction: Fabrication and biological properties of artificial tendon composite from medium chain length polyhydroxyalkanoate

**DOI:** 10.1038/s41598-024-68547-9

**Published:** 2024-08-02

**Authors:** Tulyapruek Tawonsawatruk, Anuchan Panaksri, Ruedee Hemstapat, Passavee Praenet, Kasem Rattanapinyopituk, Sani Boonyagul, Nuttapol Tanadchangsaeng

**Affiliations:** 1 Department of Orthopaedics, Faculty of Medicine Ramathibodi Hospital, Mahidol University, Thung Phaya Thai, Ratchathewi, Bangkok, Thailand; 2 College of Biomedical Engineering, Rangsit University, Lak Hok, Pathumthani, Thailand; 3 Department of Pharmacology, Faculty of Science, Mahidol University, Thung Phaya Thai, Ratchathewi, Bangkok, Thailand; 4 Department of Pathology, Faculty of Veterinary Science, Chulalongkorn University, Pathum Wan, Bangkok, Thailand

Correction to: *Scientific Reports* 10.1038/s41598-023-48075-8, published online 28 November 2023

The original version of this Article contained an error in Figure 5b, where the labels for "Fibroblast cells non-co-cultured MCL-PHA" and "Fibroblast cells co-cultured MCL-PHA" were inadvertently interchanged.

The original Figure 5 and accompanying legend appear below.

**Figure 5:**
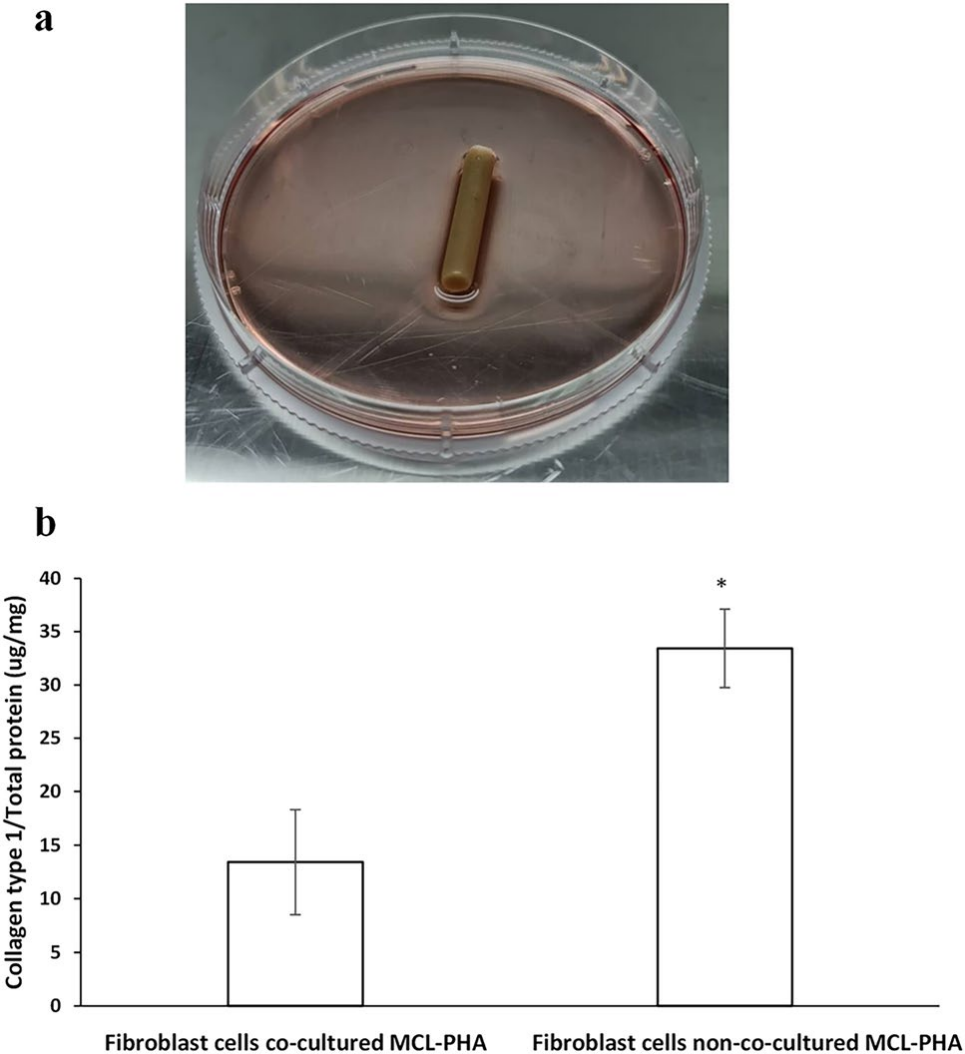
(**a**) MCL-PHA artificial tendon co-cultured with fibroblast cells. (**b**) Comparison of collagen type 1/total protein content of fibroblast cells co-cultured MCL-PHA and fibroblast cells non-co-cultured MCL-PHA (n = 3, p < 0.05, *significant, error bars of standard deviation).

The original Article has been corrected.

